# Acute Colonic Pseudo-Obstruction Secondary to Renal Calculus: A Case Report and Review of Pathophysiology

**DOI:** 10.7759/cureus.34756

**Published:** 2023-02-07

**Authors:** Maddi Gillies, Thair AlDujaili

**Affiliations:** 1 General Surgery, Goulburn Valley Health, Shepparton, AUS

**Keywords:** renal calculi, slicer software, 3d reconstruction, ogilvie's syndrome, acute colonic pseudo-obstruction

## Abstract

Acute colonic pseudo-obstruction (ACPO) is obstruction of the large bowel without a mechanical cause. The exact mechanism remains incompletely understood but is thought to result from disruption to the autonomic regulation of the colon, typically in the context of hospitalized patients with medical illness, precipitating medications, or recent surgical intervention. This paper presents an unusual case of ACPO in an ambulatory patient with a recently passed renal calculus, explores the anatomy and physiology underlying the autonomic dysfunction theory of ACPO pathogenesis in the context of the case, and provides a 3D reconstruction of the patient’s CT to illustrate the abrupt caliber change at the splenic flexure characteristic of ACPO.

## Introduction

Acute colonic pseudo-obstruction (ACPO) is an uncommon cause of large bowel obstruction with a poorly understood etiology and pathophysiology [1]. ACPO may present similarly to mechanical obstruction, making it difficult to distinguish between the two [2]. ACPO characteristically causes significant dilatation of the cecum and right colon with distal left-sided collapse, which may be demonstrated on cross-sectional imaging as an abrupt transition point at the splenic flexure without an obstructing lesion [3]. Classically, ACPO is caused by instrumentation, inflammation, hemorrhage, or other pathology in the retroperitoneum, commonly in hospitalized patients with serious illness or post-operatively in surgical patients [4]. ACPO secondary to urologic interventions including renal transplant, ablation of renal cell carcinoma, and extracorporeal shockwave lithotripsy (ESWL) for renal calculi has been reported [5-8]. This paper presents an unusual case of ACPO in an ambulatory patient with a recently passed renal calculus and reconstructed 3D views of cross-sectional computed tomography (CT) to illustrate the anatomical distribution of proximal dilatation and distal collapse characteristic of ACPO.

## Case presentation

**Figure 1:**
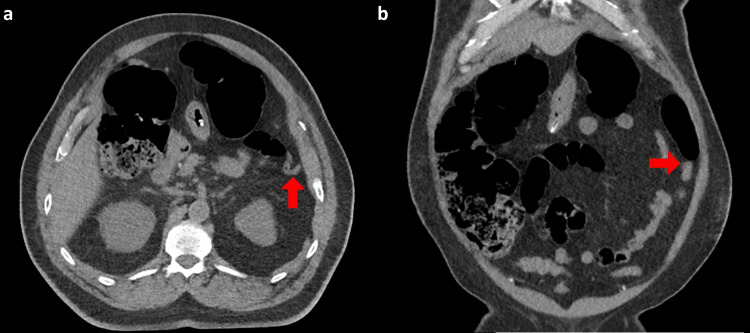
Non-contrast computed tomography scans on admission. The images show (a) axial slice demonstrating the abrupt caliber change at the splenic flexure (red arrow) and (b) coronal slice showing dilated right colon with abrupt transition to collapsed sigmoid (red arrow).

**Figure 2:**
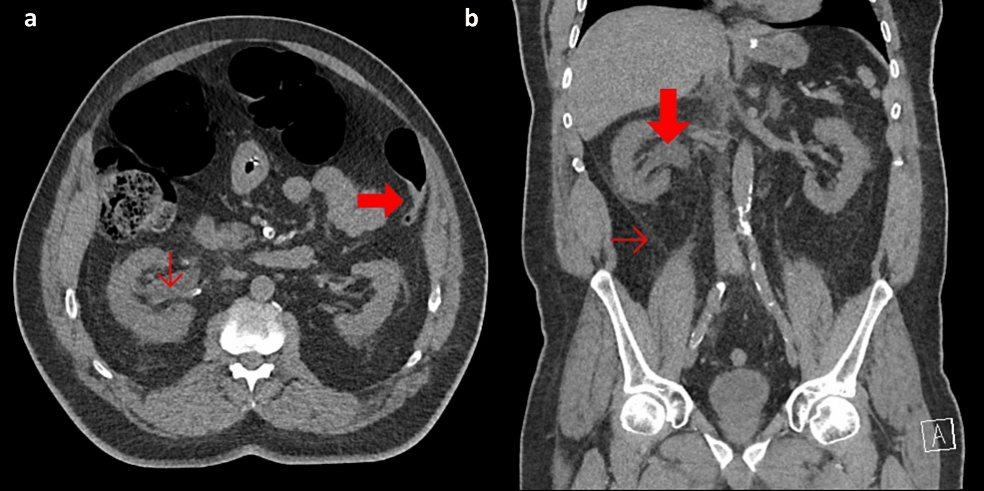
Non-contrast computed tomography scans on admission. The image show (a) axial slice demonstrating right hydroureter with surrounding perinephric stranding (thin red arrow) and re-demonstration of the abrupt transition at the splenic flexure (thick red arrow), and (b) coronal slice demonstrating right hydroureter (thick red arrow) and increased attenuation of the Gerota fascia (thin red arrow).

A 70-year-old male presented to the emergency department after three days without passing stool associated with increasing abdominal distention, vomiting, and minimal flatus. His past medical history was significant for type 1 diabetes mellitus (T1DM), ischemic heart disease with multiple coronary stents, hypertension, and chronic kidney disease. In addition, the patient described episodes of renal colic, and symptoms consistent with passing a renal calculus four days prior to presentation. On examination, the patient was hemodynamically stable and the abdomen was severely distended and tense without tenderness or rigidity. Serum hemoglobin was 134 g/L, white cell count was 15.9x10^9^/L, c-reactive protein was 110 mg/L, and estimated glomerular filtration rate (eGFR) was 26 mL/min/1.73 m^2^ (representing an acute on chronic kidney injury where baseline eGFR was 54 mL/min/1.73 m^2^), glycated hemoglobin was 7.2%, blood glucose was 15.9 mmol/L without ketones or acidosis and serum sodium was 131 mmol/L without any other electrolyte derangements. CT abdomen and pelvis revealed a dilated right colon with the cecal pole measuring 87 mm in AP projection, an abrupt caliber change at the splenic flexure, and a collapsed left colon to the rectum without an obstructing lesion (Figures 1a, 1b). In addition, right-sided hydroureter, perinephric stranding, and prominent Gerota fascia were noted, consistent with a recently passed renal calculus (Figures 2a, 2b).

**Figure 3:**
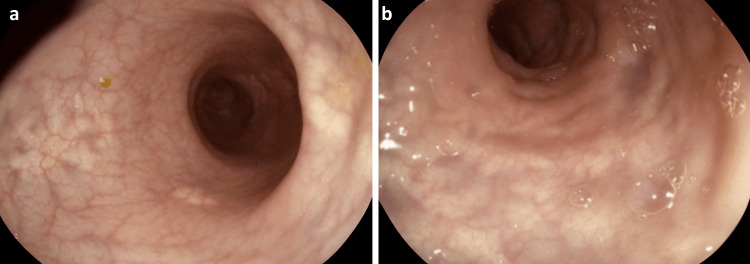
Endoscopic images showing the left colon with patchy mucosa.

The significantly abrupt transition point on CT and absence of precipitating illness raised concern for mechanical obstruction. However, without clear impending perforation indicated by a cecal diameter greater than 12 cm and in the absence of peritonism, a trial of non-operative management was commenced. A nasogastric tube was inserted for proximal decompression, intravenous fluid, and electrolyte replacement were initiated, and mobilization was encouraged with serial abdominal examination performed. While the obstruction did not resolve, a repeat CT at 72 hours showed similar findings to the admission CT, decreasing the likelihood of complete mechanical obstruction. The patient’s cardiac risk factors were relatively contraindicated cholinergic medications and management proceeded to endoscopic decompression. At colonoscopy, the mucosa appeared slightly abnormal with a patchy mottled appearance from the rectum to splenic flexure, with macroscopically normal mucosa from the splenic flexure to the cecum (Figures 3a, 3b). There was no fecal loading or distension. Random mucosal biopsies were taken from the sigmoid and rectum. Histopathology showed architecturally normal colonic mucosa without acute or chronic inflammation or evidence of mucosal ischemia. Following endoscopic decompression, the patient’s bowels began to open normally and he was discharged after a further day of observation.

**Figure 4:**
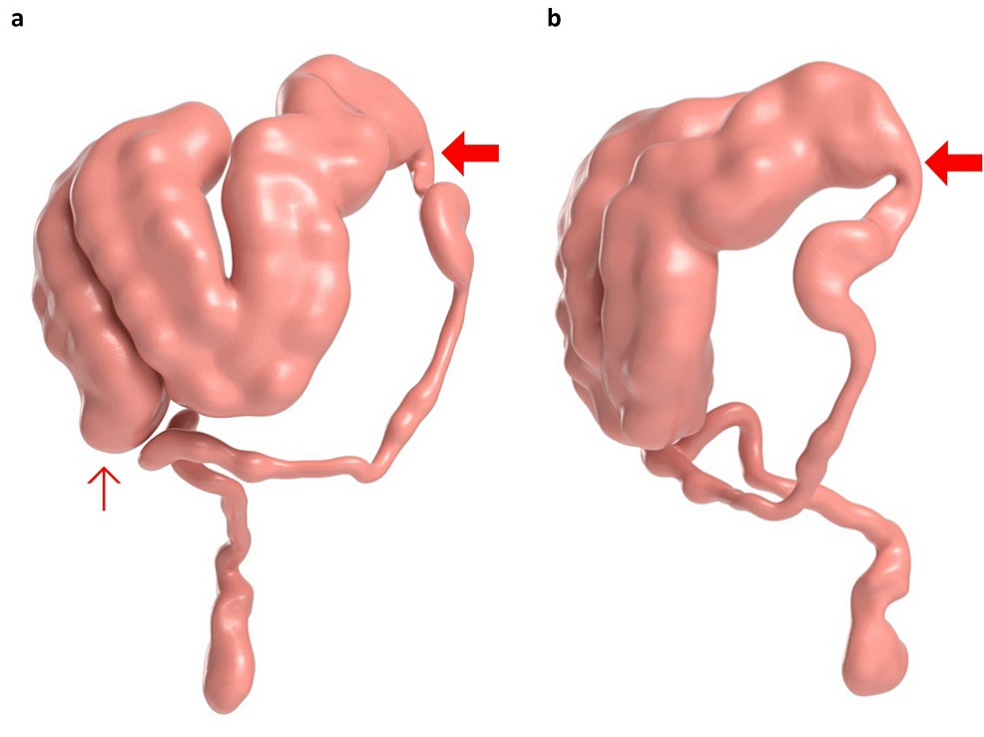
Three-dimensional reconstructions of abdominal CT. The images show (a) anterior view of colon and (b) left lateral view of colon demonstrating the dilated cecum (thin red arrow), ascending and transverse colon with abrupt caliber change about the splenic flexure (thick red arrow), and collapse of the descending and sigmoid colon.

The patient’s CT on admission was used to create a 3D mesh using pixel-wise segmentation in 3D Slicer 5.2.1 (Bucharest, Romania: Softpedia), an open-source medical image analysis software [9]. The resulting 3D mesh was textured and colored for publication using Adobe 3D Substance Painter 8.2.0 (San Jose, CA: Adobe) (Figures 4a, 4b) [10].

## Discussion

ACPO, also called Ogilvie's syndrome, is an uncommon cause of large bowel obstruction which often presents in a similar manner to mechanical large bowel obstruction [1]. Physical examination in either condition often reveals a tympanic, distended abdomen [1]. While perforation can result from either condition, mechanical obstruction is more likely to be complete, leading to perforation and peritonism. Unlike mechanical obstruction, ACPO tends to occur in conjunction with another serious illness, alongside electrolyte disturbances or post-operatively (particularly following surgery involving retroperitoneal instrumentation) [1,3]. Medications such as opiates, corticosteroids, and sedatives may also be implicated. As such ACPO is commonly seen among hospitalized patients rather than ambulatory populations [1,2]. In diagnosing ACPO, CT may demonstrate a dilated right colon, often with a transition point toward the splenic flexure, as seen in this case (Figures 1a, 1b) [2]. While this point will be without an obstructing lesion, it can be mistaken for a mechanical obstruction [2]. These similarities can present a diagnostic dilemma and a difficult decision-making process for surgeons involved in the care of such patients [2]. Non-operative management including withdrawal of medications that decrease colonic motility, nasogastric decompression, fluid resuscitation, and correction of electrolyte abnormalities along with early ambulation prove successful in 70-90% of cases [11]. When these measures do not result in resolution in 72 hours, neostigmine may be administered. In the absence of a response to pharmacologic management decompression with colonoscopy is indicated [1]. For patients in which other measures fail, or peritonism develops, surgery to raise a diverting stoma is required [12].

The exact etiology remains poorly understood, however, dysfunction in autonomic regulation of the colon appears to be central to the development of ACPO [13]. Ogilvie first described the eponymous syndrome in a 1948 paper detailing two case reports wherein the cause was found to be retroperitoneal malignancy. Ogilvie postulated that this disruption of retroperitoneal autonomic nerves resulted in “sympathetic deprivation” which was thought to decrease peristalsis [4]. Although it has since been recognized that parasympathetic input drives peristalsis, the hypothesis that autonomic dysregulation inhibits peristalsis remains true [13-15]. Increased sympathetic tone or decreased parasympathetic tone will decrease peristalsis and may contribute to an adynamic, functional obstruction of the colon or “coleus” [13]. This autonomic dysregulation theory of ACPO is supported by multiple physiologic and anatomical factors.

Response to cholinergic medication, association with circulating catecholamines, and association with autonomic neuropathy illustrate the neurophysiology of autonomic dysregulation in ACPO. Neostigmine, a cholinergic drug used in ACPO, increases available acetylcholine in parasympathetic pre- and post-ganglionic synapses leading to increased parasympathetic tone, stimulating colonic peristalsis [13,15]. Similarly, circulating catecholamines increase the sympathetic tone and thus disrupt peristalsis, increasing the likelihood of ACPO [16]. Case series have found higher incidence of acute and chronic pseudo-obstruction associated with catecholamine-secreting tumors, such as pheochromocytoma and paraganglioma [17,18]. High sympathetic tone in critical illness may also partially explain the association with ACPO [13]. Additionally, diabetic autonomic neuropathy is known to cause dysmotility along the entire gastrointestinal tract [19]. This disruption of the autonomic supply leads to gastroparesis, enteropathy, and intestinal stasis as well as acute and chronic pseudo-obstruction [19]. As such, patients with diabetes mellitus, particularly those with poor glycemic control, are at higher risk for ACPO due to autonomic dysfunction. The presented patient had longstanding T1DM with poor glycemic control, which likely contributed to the pathogenesis in this case.

The distribution of colonic dilatation with the transition point at the splenic flexure and its association with retroperitoneal pathology provides an anatomical basis for autonomic dysregulation in ACPO (Figures 1a, 1b). Sympathetic supply to the entire colon is via the thoracic and lumbar splanchnic nerves which synapse in the retroperitoneal celiac plexus about the origin of the celiac trunk [20]. Post-ganglionic sympathetic fibers then run along all midline gut branches of the aorta to supply the entire colon to the rectum [20]. While the proximal colon receives parasympathetic input from vagal fibers which enter the celiac plexus and travel alongside sympathetic fibers to supply the foregut and midgut, the distal colon receives parasympathetic fibers from the pelvic splanchnic nerves (derived from S2-S4) that supply the rectum and send fibers superiorly out of the pelvis to supply the sigmoid and descending colon [20]. Thus, the entire colon is supplied by sympathetic fibers that synapse in the retroperitoneal celiac plexus, whereas parasympathetic supply is derived from celiac plexus vagal fibers only until the splenic flexure, at which point the pelvic splanchnic fibers provide this function. This distribution of autonomic nerve supply is consistent with the transition point at the splenic flexure often seen in ACPO, implicating autonomic dysfunction in the pathogenesis of ACPO. Moreover, ACPO has commonly been associated with retroperitoneal pathology [13]. In this case, history indicated a renal calculus with perinephric fat stranding on CT, suggesting an inflammatory process in the right retroperitoneal space (Figures 4a, 4b). While previous case reports have demonstrated ACPO secondary to various urological interventions, such as renal transplantation and extracorporeal shockwave lithotripsy (ESWL) for renal calculi, this is the case that documents ACPO in association with renal calculi without the additional retroperitoneal trauma of ESWL [5-8]. While the pathophysiology of ACPO is clearly multifactorial, it could be concluded that inflammation in the retroperitoneal has the potential to disrupt the retroperitoneal autonomic supply and predispose to ACPO in a patient with significant risk factors for autonomic dysfunction, such as long-standing T1DM with poor glycemic control.

## Conclusions

This report has presented a novel case of ACPO in association with renal calculus. While the pathophysiology is not entirely clear, this case provides insight into the anatomy and physiology of autonomic disruption in ACPO. Moreover, this case illustrates that ACPO should be suspected even in ambulatory patients who have any evidence of retroperitoneal pathology in the absence of evidence for mechanical obstruction, especially in those who have risk factors for autonomic dysfunction such as diabetes mellitus.
